# Two Birch Species Demonstrate Opposite Latitudinal Patterns in Infestation by Gall-Making Mites in Northern Europe

**DOI:** 10.1371/journal.pone.0166641

**Published:** 2016-11-11

**Authors:** Mikhail V. Kozlov, Anna Skoracka, Vitali Zverev, Mariusz Lewandowski, Elena L. Zvereva

**Affiliations:** 1 Section of Ecology, Department of Biology, University of Turku, Turku, Finland; 2 Population Ecology Laboratory, Faculty of Biology, Adam Mickiewicz University, Poznan, Poland; 3 Department of Applied Entomology, Faculty of Agriculture, Biotechnology and Landscape Architecture, Warsaw University of Life Sciences—SGGW, Warsaw, Poland; Nanjing Agricultural University, CHINA

## Abstract

Latitudinal patterns in herbivory, i.e. variations in plant losses to animals with latitude, are generally explained by temperature gradients. However, earlier studies suggest that geographical variation in abundance and diversity of gall-makers may be driven by precipitation rather than by temperature. To test the above hypothesis, we examined communities of eriophyoid mites (Acari: Eriophyoidea) on leaves of *Betula pendula* and *B*. *pubescens* in boreal forests in Northern Europe. We sampled ten sites for each of five latitudinal gradients from 2008–2011, counted galls of six morphological types and identified mites extracted from these galls. DNA analysis revealed cryptic species within two of six morphologically defined mite species, and these cryptic species induced different types of galls. When data from all types of galls and from two birch species were pooled, the percentage of galled leaves did not change with latitude. However, we discovered pronounced variation in latitudinal changes between birch species. Infestation by eriophyoid mites increased towards the north in *B*. *pendula* and decreased in *B*. *pubescens*, while diversity of galls decreased towards the north in *B*. *pendula* and did not change in *B*. *pubescens*. The percentage of galled leaves did not differ among geographical gradients and study years, but was 20% lower in late summer relative to early summer, indicating premature abscission of infested leaves. Our data suggest that precipitation has little effect on abundance and diversity of eriophyoid mites, and that climate warming may impose opposite effects on infestation of two birch species by galling mites, favouring *B*. *pendula* near the northern tree limit.

## Introduction

During the past decade, the latitudinal variation in invertebrate herbivory has attracted an increasing attention from ecologists [1–9]. However, the majority of the published studies aimed at identification of general macroecological patterns, while much less attention had been paid to the sources of variation in the relationships between herbivory and latitude and to uncovering mechanisms behind these relationships.

Plant-herbivore interactions often differ among feeding guilds of herbivores [9–11]. In particular, invertebrates feeding within plant tissues (stem/root borers, leaf miners and gall-makers) not only can manipulate the quality of plant tissues for their own benefit, but also are protected by their hosts from direct impacts of abiotic environments, to which externally feeding defoliators are exposed directly. As a result, endophagous species may differ in their responses to biotic and abiotic factors from externally feeding defoliators [12, 13].

The recent study of global latitudinal patterns in herbivory, i.e. of variations in plant losses to animals with latitude, found that gall-makers demonstrate unusual relationships with latitude and climate: their abundance, in contrast to defoliators, increased with latitude [6]. In tropical and temperate climates gall-making insects were found to have higher population densities in xeric habitats than in mesic habitats [14]. On the other hand, the incidence of galling on oak leaves in California increased with an increase in precipitation [13]. Thus, it is likely that geographical patterns in woody plant damage imposed by gall-forming herbivores are primarily driven by factors other than temperature, in particular by precipitation and humidity. Consequently, gall-forming invertebrates may respond to the ongoing climate change in a different way than defoliators do.

Northern Europe offers an unique opportunity for untangling the effects of precipitation from the effects of temperature and other environmental drivers of latitudinal patterns in herbivory. An average rainfall on the Atlantic coast of Norway exceeds the rainfall in north-eastern Europe by a factor of four or more, whereas precipitation at the southern border of boreal forests (approx. 60° N) in Finland and NW Russia is less than two times as high as at the northern tree limit (approx. 70° N) [15]. Thus, if plant infestation by gall-makers is driven by precipitation, this pattern allows us to expect that the differences in birch infestation by gall-making mites between coastal and continental regions will be higher that the differences in incidence of leaf galling between northern and southern ends of latitudinal gradients.

The coexisting herbivore species often demonstrate different latitudinal patterns [3]. Still, ecological studies of herbivory generally combine all species that produce leaf galls. This is often unavoidable, because identification of a large number of samples that are routinely collected for ecological projects requires taxonomic expertise, time and resources that all are generally in short supply. To partially overcome this problem, for the present study we selected eriophyoid mites (Acari: Eriophyoidea), because it is commonly assumed that their galls, which are so distinctive in appearance, can be used for species identification [16]. Therefore, the percentages of leaves with different types of galls have been expected to provide reliable estimates of population densities of these herbivores [17–19].

Eriophyoid mites often impose severe damage on woody plants, e.g. on birches, leaves of which can be nearly completely covered by felt galls (erinea) or bear hundreds of warty, pouch, or cephaloneon galls [16]. This damage often reduces plant fitness [20, 21]; furthermore, the heavily galled leaves of common alder attacked by *Eriophyes laevis* (Nalepa) were observed to abscise earlier than leaves with low or moderate gall densities [22]. In highly seasonal environments, premature abscission of damaged leaves may result in underestimation of plant losses to herbivory [23]; but the magnitude of this bias and its possible impact on the detected latitudinal patterns in herbivory remain unknown.

The objective of our study was to investigate geographical variation in the abundance and diversity of gall-makers in boreal forests and elucidate environmental factors shaping this variation. For this purpose we recorded the occurrences of different types of galls formed by eriophyoid mites on leaves of two species of birches in the course of an extensive monitoring program that had been implemented during 2008–2011 in Northern Europe, from 59 to 69° N and from 10 to 60° E (for other outcomes of this program consult [3, 4, 24]). We tested the hypotheses that: (1) the intensity of infestation and diversity of galls change with latitude; (2) the intensity of infestation differs among geographical regions, study years, birch species and gall types; (3) geographical variation in the intensity of infestation is explained by precipitation better than by temperature; (4) galled leaves abscise prematurely, distorting latitudinal patterns deducted from samples collected late in the growing season.

## Material and Methods

### Study area and sampling sites

**Fig 1:**
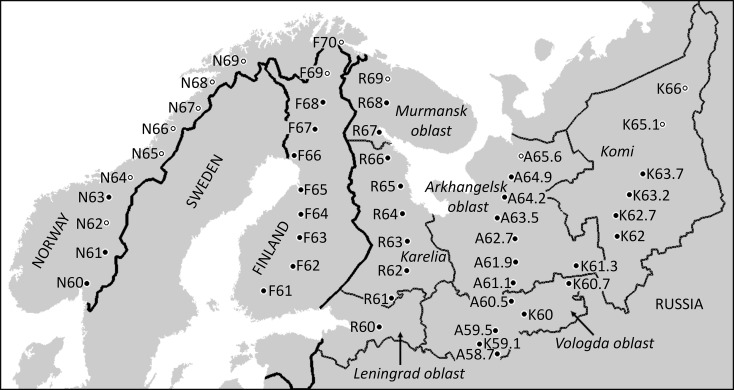
Study area and study sites. Name of each study site contains the code of the gradient and the approximate latitude (for the coordinates of study sites see S1 Table). Open circles denote study sites where the samples were collected only from *Betula pubescens*. Reprinted from [3] under a CC BY license, with permission from John Wiley and Sons, original copyright 2013.

Sampling was conducted along five latitudinal gradients which were between 750 and 1300 km in length (Fig 1: N, from Olso to Andselv, Norway; F, from Turku to Nuorgam, Finland; R, from St. Petersburg to Murmansk, Russia; A, from Vologda to Arkhangelsk, Russia; and K, fom Vologda to Inta, Russia). All gradients were located in Scandinavian and Russian taiga, an ecoregion within the taiga and boreal forests biome. Typical coniferous forests of this ecoregion are dominated by Scots pine (*Pinus sylvestris* L.) or Norway spruce (*Picea abies* (L.) Karst.) but also have significant numbers of downy and white birches (*Betula pubescens* Ehrh. and *B*. *pendula* Roth, respectively). No permits were required for the described study, which did not involve endangered or protected species and was conducted outside the protected areas and therefore complied with all relevant regulations.

The sampling sites were selected in forests typical for each locality; care was taken to select a representative site where both *B*. *pubescens* and *B*. *pendula* grow naturally. This was impossible in some areas, and therefore in 13 of the 50 sampling sites we collected samples only from *B*. *pubescens* (Fig 1; S1 Table). The northernmost site in the F gradient was excluded from the analysis, because during the study year the birches at this site were completely defoliated by the larvae of autumnal moth, *Epirrhita autumnata* (Bkh.), and no leaves were available to quantify infestation by gall mites. Each site was sampled twice a year. The R gradient was sampled each year (23–29 July and 21–25 August 2008, 24–29 June and 24–28 August 2009, 22–27 June and 29 July– 2 August 2010, 12–16 June and 10–14 August 2011), while all other gradients were each sampled during one year (N gradient: 29 June– 2 July and 27–30 August 2011; F gradient: 25–26 June and 2–4 September 2008; A gradient: 16–18 June and 7–9 August 2010; K gradient: 18–20 June and 1–3 September 2009). We estimate that less than 1% of new leaves (i.e., apical leaves of long shoots) were formed by our study trees between the first and the second sampling. The second sampling was conducted 15–50 days before the beginning of seasonal leaf fall.

Climatic data (average temperatures for January and July and annual precipitation) were obtained using New_LocClim [25].

### Sampling and processing

Mature trees (generally aged 20 years or more) with lower branches that can be reached from the ground (i.e. within 2 m height) were selected on a ‘first found, first sampled’ basis. One branch about 50 cm in length (with approximately 80 leaves) was collected from each of the five trees of each birch species at each site on each sampling date. The sampled trees were not tagged and therefore early and late summer samples were generally collected from different trees.

In the laboratory, the leaves on each branch were counted, and each leaf was carefully inspected for damage caused by eriophyid mites. A total of 121,876 leaves were examined. In 2008, we only counted galls of type 5 which have been presumed to be most common in our study region; but the discovery of the variety of the gall types in 2008 prompted us to change the methodology of future censuses. From 2009–2011 we distinguished six types of galls (S1 Fig) and counted the numbers of leaves containing these galls in each sample. Intensity of leaf damage was measured by percentages of leaf area covered by felt galls (types 1 and 2) and by numbers of galls of all other types per infested leaf (S1 Data).

For morphological examination, eriophyoid specimens were brought out from representative samples of galls, mounted in Keifer’s booster [26] and heated at about 90° C for few minutes in the purpose of clearing. Additionally, 140 permanent slides were done by mounting mite specimens in Berlese medium according to a standard protocol [27]. All specimens were studied using a phase-contrast microscope and identified to the genus using [28]. Species-level identities were established by comparing the specimens with primary descriptions of relevant taxa [29–34]. Voucher specimens of galls and mites are deposited in the Department of Animal Taxonomy and Ecology, Adam Mickiewicz University in Poznań.

For molecular identification, mites were transferred into a DNase-free 0.5 ml polyvinyl tubes containing 5 μ l of Proteinase K (20 mg/ml), crushed using a clean steel needle and 100 μl of 6% Chelex® solution was added. Further procedure followed method described in [35]. A partial sequence of the cytochrome oxidase subunit I (COI) of mitochondrial DNA was amplified by PCR with degenerate primers: bcdF01 and bcdR04 [36] and Qiagen Multiplex PCR Kit. PCRs were conducted in 25 μl reaction volumes according to the standard protocol with 7.5 μl DNA template. PCR products were purified using Exonuclease I and Shrimp Alkaline Phosphathase enzyme mix (Thermo Scientific, USA) and directly sequenced in forward direction using the BigDye Terminator v3.1 chemistry on an ABI3730 Genetic Analyzer (Applied Biosystems, USA) in the DNA Sequencing and Oligonucleotide Synthesis Laboratory IBB PAS (Warsaw, Poland).

### Data analysis

Trace files were checked and edited using MEGA version 6 [37]. Basic Local Alignment Search Tool (BLAST) search of the sequence was performed on the National Center for Biotechnology Information (NCBI) GenBank database to confirm the identity of obtained sequences with sequences of Eriophyoidea. Subsequently the sequences were aligned by CLUSTALW with default gap weighting parameters. Alignment of the COI sequences covering 605 bp was translated into amino acids to exclude the presence of pseudogenes or stop codons. The uncorrected pairwise genetic distances (%) were calculated, with standard error estimates obtained using a bootstrap procedure (1,000 replicates). All computations were made using MEGA version 6 [37]. All sequences have been deposited in GenBank under the accession numbers KU315228–KU315238.

Distributions of the percentages of galled leaves among branches and among study sites were greatly skewed: no galls were found in 791 of 1427 collected branches (55.4%), and all leaves in four branches were galled. Therefore we used non-parametric methods in the analysis of these data. We calculated Spearman rank correlation coefficients to explore the relationships between the percentage of infested leaves in a sample and the intensity of infestation of an individual leaf and used Kruskal-Wallis test to compare the percentages of galled leaves between early and late season censuses, birch species, geographical regions and study years.

As a measure of diversity, we used the Shannon’s index (H = -Σ[p_i_ × ln p_i_], where p_i_ = the proportion of leaves with *i*th gall type of the total number of leaves with galls of eriophyoid mites), calculated from the site-specific numbers of galled leaves. For sites with no galls H was set to 0.

Latitudinal patterns were quantified by calculating Spearman rank correlation coefficients between in the percentage of the infested leaves or diversity of gall types and the latitude of the study site. To uncover mechanisms behind the latitudinal variation, we correlated the percentage of the infested leaves and diversity of gall types with climatic variables. For the percentage of the infested leaves, individual correlations with latitudes of sampling sites (S2 Table) refer to gall type × gradient × study year × sampling date × birch species combination. Sources of variation in the relationships of the percentage of galled leaves with latitude were explored using meta-analysis. To calculate effect sizes (ES), individual correlation coefficients were *z*-transformed and weighted by their sample size using the standard procedure in the MetaWin program [38]. In our study the positive ES values indicate increases in infestation with latitude. If the number of ES in an individual group was nine or less, a bootstrap estimate of the 95% confidence interval (*CI*_*95*_) was used. The effect was considered statistically significant if its *CI*_*95*_ did not include zero. Meta-analysis was performed using random effects categorical models that compared ES between censuses, birch species and gall types. The variation in the ES among the classes of categorical variables was explored by calculating the heterogeneity index (*Q*_B_) and testing it against the *χ*^*2*^ distribution [39].

## Results

### Identities of mites forming different types of galls

Four of six species of mites morphologically identified from our samples were found in more than one type of gall (Table 1). Thus, the exact correspondence between the gall type and mite species cannot be established; however, we still found that felt galls (types 1–2) almost exclusively contained *Acalitus longisetosus* (Nalepa) and *Acalitus rudis* (Canestrini), while warty galls (type 5) contained *Eriophyes leionotus* (Nalepa). These three common mite species were identified from both *B*. *pendula* and *B*. *pubescens*, whereas three other species (*Aceria fennica* (Lindroth), *A*. *lissonota* (Nalepa) and *A*. *vinosa* Roivainen) were found on *B*. *pubescens* only. The data on the confirmed occurrence of morphologically identified mite species in each of our study sites are summarized in S1 Table and the results of density estimations can be found in S1 Data.

**Table 1:** Correspondence between gall types, morphologically identified species of mites and mite genotypes identified by DNA analyses.

Species	Numbers of mite samples^a^, identified on the basis of morphological characters from each gall type (in parentheses: mite genotype)^b^					
	#1	#2	#3	#4	#5	#6
*Acalitus longisetosus*	11 (A)	10 (B)	2 (-)	-	-	-
*Acalitus rudis*	1 (C)	12 (C)	2 (C)	-	1 (-)	
*Aceria fennica*	-	1 (D)	1 (D)	-	-	
*Aceria lissonota*	-	-	-	-	-	3 (E)
*Aceria vinosa*	-	-	-	-	-	2 (-)
*Eriophyes leionotus*	-	-	2 (F)	2 (F)	62 (G)	13 (H)

^a^A sample generally included several mites extracted from 4–5 galls, and several DNA sequences from each morphologically identified mite species by gall type combination.

^b^Missing genotype data indicate that sequence was impossible to obtain due to shortage of material.

The barcoding of mites based on the mitochondrial DNA COI sequences revealed the existence of eight distinct genotypes (p-distance from 10.8 to 37.3%; S3 Table) within five morphological species (Table 1). Most importantly, we discovered two genotypes of *A*. *longisetosus* and three genotypes of *E*. *leionotus*, associated with different types of galls. The comparisons of the uncorrected pairwise genetic distances between genotypes within morphologically defined species with the distances within these genotypes (more than tenfold) suggests the inter-specific level of genetic differentiation between mite genotypes associated with different types of galls. The taxonomic treatment of these findings will be published elsewhere.

### Frequencies of galled leaves and numbers/areas of galls

At the level of individual trees, frequencies of leaves with all types of galls strongly correlated with average areas (gall types 1–2) or numbers (gall types 3–6) of galls per leaf (*r*_S_ = 0.82…0.95, *n* = 19…318 trees, *P* < 0.0001). Therefore the subsequent analyses were restricted to the frequencies of the infested leaves.

### Differences between early and late summer censuses

Leaves infested by *A*. *longisetosus* (gall type 1) and primarily by *E*. *leionotus* (types 4 and 6) were equally frequent in samples collected in early and late summer. Leaves with all other types of galls were more frequent during the early summer (Table 2). Consequently, the overall percentage of galled leaves in samples collected in early summer was significantly higher than in samples collected in late summer (Table 2). However, when all types of galls were pooled, the correlations between the percentage of galled leaves and latitude did not differ between early and late summer censuses (*Q*_B_ = 0.02, *df =* 1, *P* = 0.89).

**Table 2:** Differences in abundance of eriophyoid mite galls between two censuses and between two birch species.

Gall type	Percentage of infested leaves		Differences between early and late summer		Percentage of infested leaves in early summer		Differences between birch species	
	Early summer	Late summer	*χ*^*2*^	*P*	*B*. *pendula*	*B*. *pubescens*	*χ*^*2*^	*P*
1	0.55	0.59	0.97	0.22	0.00	0.96	32.9	<0.0001
2	2.41	1.99	8.58	0.0034	1.87	2.84	0.25	0.62
3	0.11	0.01	14.4	0.0001	0.02	0.14	5.02	0.03
4	0.22	0.09	3.24	0.07	0.38	0.18	0.30	0.58
5	4.26	2.82	5.59	0.02	8.61	1.36	98.9	<0.0001
6	2.99	3.12	0.04	0.84	0.12	4.61	45.7	<0.0001
**All types pooled**	10.10	8.18	10.5	0.0012	10.51	9.78	2.72	0.10

### Differences among geographical gradients and study years

The percentage of galled leaves did not differ either among the latitudinal gradients (*χ*^2^ = 0.72, *df =* 3, *P =* 0.87), between the coastal (N) gradient and the remaining (continental) gradients (*χ*^2^ = 0.31, *df =* 1, *P =* 0.58) or among study years (*χ*^2^ = 0.95, *df =* 2, *P =* 0.62). When the last comparison was restricted to the R gradient that was sampled during all study years, the variation remained non-significant (*χ*^2^ = 2.99, *df =* 2, *P =* 0.22). Similarly, latitudinal patterns in the percentage of galled leaves did not differ among the geographical gradients (*Q*_B_ = 4.69, *df =* 3, *P* = 0.20) and study years (*Q*_B_ = 0.79, *df =* 2, *P* = 0.64). In contrast, Shannon index demonstrated marginally significant variation among gradients (*χ*^2^ = 7.52, *df =* 3, *P =* 0.06), from 0.148 in the A gradient to 0.400 in the K gradient.

### Differences among gall types

**Fig 2:**
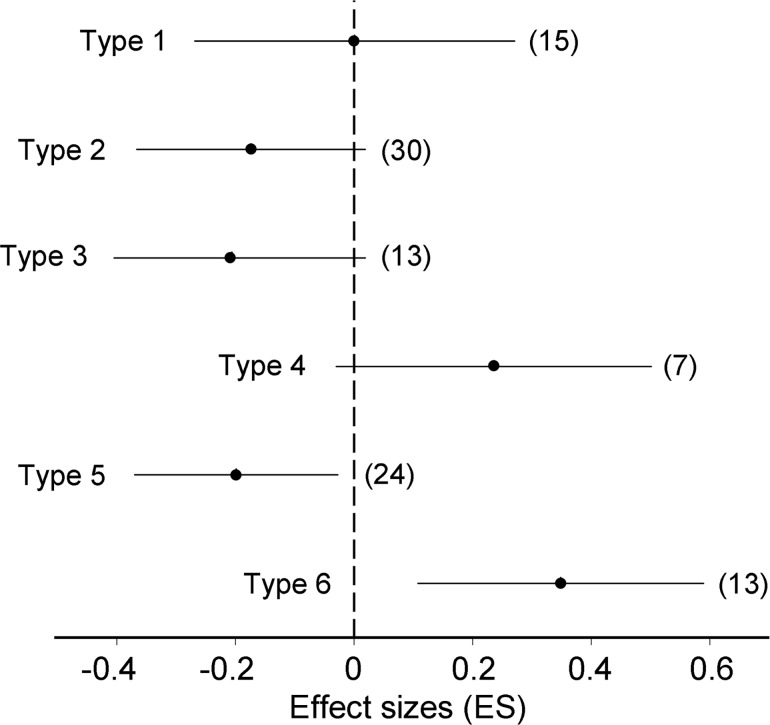
Variation in latitudinal patterns of the percentage of birch leaves infested by different types of galls (results of meta-analysis). Negative ES values indicate decrease in abundance with latitude. Horizontal lines denote 95% confidence intervals; sample sizes are shown in brackets. For the description of gall types, consult S1 Fig; for correspondence between gall types and species/genotypes of mites, consult Table 1.

We have obtained 102 correlation coefficients between the latitudes of study sites and the frequencies of the infested leaves for gall type × gradient × study year × sampling date × birch species combinations. Among these coefficients, only 12 were significant at the probability level *P =* 0.05 (S2 Table). In general, the percentages of leaves with different gall types did not change with latitude: an overall effect was not significant (ES = 0.02, *CI*_95_ = -0.07 to 0.12). However, the occurrences of different types of galls demonstrated different relationships with latitude (*Q*_B_ = 23.7, *df =* 5, *P* = 0.001). When the data from two birch species were combined, the occurrence of gall type 5 significantly increased towards the North, the occurrence of gall type 6 decreased, and the occurrences of other types of galls showed no significant relationships with latitude (Fig 2).

### Differences between birch species

Leaves with galls of the type 5 were considerably more frequent on *B*. *pendula* than on *B*. *pubescens*. Leaves with galls of the types 1, 4 and 6 showed the opposite pattern, while the remaining two types of galls (2 and 4) were equally abundant on both species of birch. As a result, the two birch species did not differ in the overall percentage of leaves galled by eriophyoid mites (Table 2).

**Fig 3:**
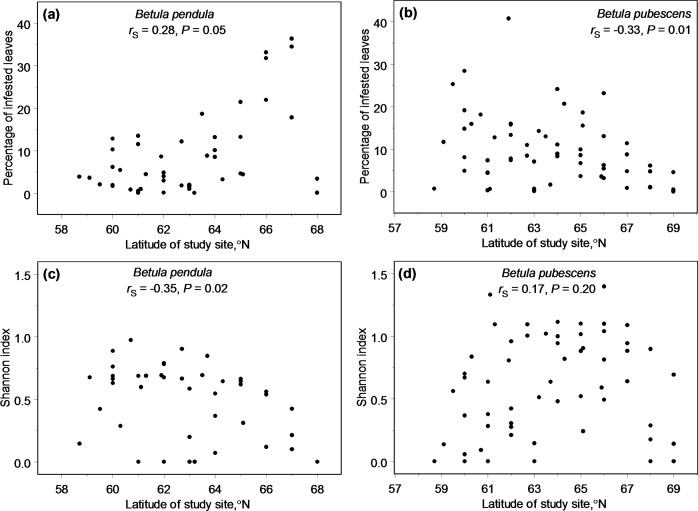
**Changes in overall proportion of leaves infested by eriophyoid mites (a, b) and in the diversity of gall types (c, d) on *Betula pendula* (a, c) and *B*. *pubescens* (b, d) along latitudinal gradients during 2009–2011.** For positions of gradients see Fig 1.

When the data were combined across gall types, the overall percentage of leaves infested by eriophyoid mites increased with latitude in *B*. *pendula* and decreased in *B*. *pubescens* (Fig 3A and 3B). Consequently, infestation increased with temperature in July in *B*. *pubescens* (*r*_S_ = 0.37, *n* = 60 sites, *P* = 0.004) and decreased in *B*. *pendula* (*r*_S_ = -0.29, *n* = 47 sites, *P* = 0.05). The percentage of infested leaves decreased with an increase in precipitation in *B*. *pubescens* (*r*_S_ = -0.33, *n* = 60, *P* = 0.02) but not in *B*. *pendula* (*r*_S_ = -0.03, *n* = 47, *P* = 0.84).

The Shannon index based on numbers of leaves with different types of galls demonstrated poleward decrease in *B*. *pendula* but did not change with latitude in *B*. *pubescens* (Fig 3C and 3D). The latitudinal pattern in the diversity of mite galls in *B*. *pendula* was explained by temperature in July (*r*_S_ = 0.39, *n* = 47, *P* = 0.007) but was independent from precipitation (*r*_S_ = 0.14, *n* = 47, *P* = 0.34). In contrast, the diversity of galls in *B*. *pubescens* decreased with an increase in precipitation (*r*_S_ = -0.34, *n* = 60, *P* = 0.007) but was independent of temperature in July (*r*_S_ = -0.06, *n* = 60, *P* = 0.62).

## Discussion

### Usability of gall characteristics for ecological studies

At the planning stage of this study we believed that, in accordance with a frequently expressed opinion [16, 18], counting of galls of different types is sufficient for reliable evaluation of population densities of different species of eriophyoid mites. This approach had been earlier used in ecological studies: for example, different types of galls observed on birch leaves were unequivocally attributed to different species of mites [17, 19, 40]. Several other studies on galling eriophyoid mites associated with deciduous trees also identified mite species using the characteristics of galls rather than of mite specimens [21, 22, 41, 42].

Keeping in mind that birches in our study region host 12 species of eriophyoid mites, and among them nine species have been recorded as gall-makers [43, 44], at the beginning of data collection, M.V.K. and E.L.Z. classified the galls found on birch leaves into eight morphotypes. Later on, microscopic examination of two gall types revealed that they were produced by insects, and therefore only six gall types were attributed to mites (S1 Fig). However, the correspondence between gall type and mite species appeared ambiguous: four of six morphologically identified mite species were each found in 2–4 types of galls and, vice versa, five of six gall types were found to contain 2–3 species of mites.

Importantly, *E*. *leionotus* has been reported to produce two types of galls, erineum and cephaloneon [43, 44], and our morphology-based identifications of mites supported this opinion. At the same time, some sources [45] illustrated for this species only erinea (our type 6) formed along the midvein on the underside of birch leaves, but attributed warty galls (our type 5) to *Cecidophyopsis betulae* (Nalepa), which we did not discover in any of the examined galls. In contrast, other researchers [17] attributed warty galls (our type 5) to *E*. *leionotus*, in accordance with the primary description of this species [30].

The largest overlap between the occurrences of different mite species was found in the white felt galls (erineum), which equally often contained *A*. *longisetosus* and *A*. *rudis*, while red felt galls contained almost exclusively *A*. *longisetosus*. Indeed, the tiny size and structural simplicity of eriophyoid mites hinder their taxonomic identification and differentiation between morphologically related species [46]. Recently, advents in DNA barcoding have been successfully employed in studies of eriophyoid mites and led to discovery of many morphologically cryptic species with inter-species divergence in mtDNA COI ranging from 11% to 28% [36, 47]. Genetic analyses showed that white and red felt galls contained different genotypes of *A*. *longisetosus* but the same genotype of *A*. *rudis*. Of course it is impossible to decide whether the galls, especially ‘open galls’ like erinea, were actually induced by the mites found within the galls, since eriophyoid species can walk between galls and are regular guests of galls caused by other species [48, 49]. Still these results are somewhat disappointing, because they do not allow deducting conclusions on the abundances of individual species of galling mites from the data on frequencies of different types of galls. However, the discovery of different genotypes (which, potentially, represent cryptic species) within *A*. *longisetosus* and *E*. *leionotus* significantly improved the correspondence between gall types and responsible organisms. Furthermore, the total proportion of leaves infested by eriophyoid mites, which is potentially linked with plant performance [20], is not affected by this problem.

Our results stress the need in taxonomic identification of mite specimens, based on their morphology and DNA, for any ecological study that involves counting of their galls, because colour, shape, structure and position of galls may be influenced by complex relationships between mite genotype, host genotype and environment [20]. Our data clearly indicate that a gall’s appearance cannot be used to reliably identify the mite concerned, at least for the species inhabiting birch leaves. Moreover, morphological and DNA analyses of specimens found in galls should include a large number of galls of a given type and a large number of leaves. Further experimental studies of gall formation are needed to associate gall-makers with certain types of galls and identify characters that can be used to differentiate galls on birch leaves.

### Differences between birch species

The two studied species of birches are closely related to each other [50] and can produce hybrids [51]; pronounced morphological variation within both species makes them sometimes hard to differentiate [52, 53]. However, for the present study we sampled only those trees that had a suite of morphological characters allowing their unequivocal attribution to either *B*. *pubescens* or *B*. *pendula*.

The two studied birch species have a very wide climatic tolerance, but *B*. *pendula* extends further south than *B*. *pubescens* in Europe and Asia, whereas the distribution of *B*. *pubescens* extends more northerly and easterly than that of *B*. *pendula*. In general, *B*. *pendula* prefers dry, sandy soils, while *B*. *pubescens* is more common on wet, poorly drained sites such as clay soils and peat bogs [54]. However, in many habitats these species grow next to each other.

The majority of literature reports on herbivores feeding on birches do not distinguish between *B*. *pendula* and *B*. *pubescens*. However, a number of cases have been recorded in which closely related insect species have a preference for one of these birch species [3, 54]. The differences in herbivore communities between *B*. *pendula* and *B*. *pubescens* may result from differences in phenology [55] or biochemistry [56] of these birch species, but this question remains completely unexplored. The detected differences in the occurrence of different types of eriophyoid galls on two birch species further indicate that the identification of a host plant as ‘*Betula* sp.’, that is commonly reported in ecological studies of eriophyoid mites [19, 57, 58] and herbivorous insects [54], is insufficient, and that combination of data on mite galls collected from two birch species (as it had been done in [17]) may easily yield misleading results.

The difference in the direction of latitudinal changes in the abundance and diversity of eriophyoid galls between two species of birches is the most intriguing finding of our study. The poleward increase in the overall percentage of galled leaves in *B*. *pendula* is exceptional: the increase in herbivory with an increase in latitude had only rarely been reported, especially from high-latitude regions [2], where the damage of woody plant leaves generally decreases towards the poles [3, 5, 6]. Furthermore, the damage of *B*. *pendula* and *B*. *pubescens* by defoliating insects in Fennoscandia followed the same pattern [1]. On the other hand, herbivores from different feeding guilds may show opposite latitudinal patterns on the same host plant, as was demonstrated for seed and leaf herbivory on the perennial herb *Ruellia nudiflora* (Engelm. & A.Gray) Urb. in North America [9].

The discovered differences in latitudinal patterns in infestation of two birch species result from differential preferences of eriophyoid mites producing different types of galls on these birch species. On *B*. *pendula*, 81.9% of all galled leaves contained warty galls (type 5), the abundance of which increased with latitude. In contrast, on *B*. *pubescens* this gall type is infrequent (13.9%), while the abundance of the most common (47.1%) gall type 6 decreased with latitude. It appeared indeed strange that these two types of galls (5 and 6), in spite of discovered ecological differences, predominantly contained the same species of gall mite, *E*. *leionotus*. However, our DNA analysis strongly suggest that, in line with the opinion by Amrine [44], these two types of galls are actually induced by different, albeit morphologically similar, species which are so far combined under the name *E*. *leionotus*.

### Weather conditions and abundance of galling mites

Previous examinations of temperature and humidity (measured by water stress, rainfall or soil moisture) as factors affecting abundance of eriophyoid galls led to variable results with ambiguous linkage to climate [13, 18, 19]. For example, no correlations were found between the occurrence of the birch-galling eriophyoid mites and precipitation or temperature, except for relation of felt galls to rainfall [19]. Consistently with these studies, our data provide equivocal support for the hypothesis that abundance and diversity of eriophyoid mites in boreal forests of Northern Europe are primarily driven by precipitation. First, we did not find the differences in the occurrence of mite galls between the Atlantic coast (N gradient) and continental (R, A and K) gradients, in spite of fourfold [15] differences in precipitation between these regions. Second, in about half of the situations, temperature explained variation in characteristics of galling mite communities better than precipitation. Furthermore, the differences in infestation of two birch species by different gall types, discovered in this study, suggests that weather conditions may differently affect abundances of mite species on *B*. *pendula* and *B*. *pubescens*.

At the same time, in contrast to sap feeding and leaf mining insects [3, 4], eriophyoid mites demonstrated no variation in abundance between study years. In combination with earlier findings on the absence of changes in prevalence, density, or intensity of galls caused by eriophyoid mites on *Salix arctica* Pall. leaves in response to manipulations of temperature and water supply [59], this result suggests that eriophyoid mites cannot fully exploit the opportunities offered by favourable weather conditions during certain growth seasons and therefore quick climate-driven changes in plant infestation by gall mites can hardly be expected.

### Premature abscission of galled leaves

All mites feeding on birches in our study region continue to produce galls until the plant starts to pull back the nutrients from leaves in the autumn [60]. Thus, in the late summer we may expect to find higher percentage of galled leaves compared to early summer, provided that mites colonize new leaves during the season. Although this behaviour has not been confirmed experimentally, the patterns of gall distributions within the shoots [18, 22, 61] suggest that movement of galling mites between leaves and formation of new galls during season are likely. On the other hand, heavy galled leaves of common alder fall down earlier than their intact or slightly galled neighbours [22].

Our comparison between the percentages of galled leaves in samples collected in early and late summer suggests that at least 20% of birch leaves, which were infested by gall mites in early summer, had abscised by the late summer, 15–50 days before the typical beginning of the seasonal leaf fall. Actually, this estimate is rather conservative: if we assume that infestation of new leaves did occur between our censuses, then the percentage of prematurely abscised leaves is larger than 20%. Thus, the late summer sampling greatly underestimates the levels of birch foliage infestation by eriophyoid mites; however, latitudinal patterns in birch infestation were not distorted by premature abscission of galled leaves.

We conclude that, in addition to the direct effects on plant performance, mediated in particular by large reductions in photosynthesis [21], infestation by eriophyoid mites imposes additional costs on birches through the loss of opportunities for further photosynthesis. A reduction in leaf life span by 15–50 days, i.e. of the substantial part of the growth period, which in our study region lasts 110–140 days, further decreases the supply of assimilates to the local and/or plant-wide sinks.

## Conclusions

Our study is the first to explore large-scale geographical variation in abundance of eriophyoid mites and diversity of their galls. We demonstrated that, in general, the levels of infestation of birch leaves by eriophyoid gall mites are independent of latitude in boreal forests in Northern Europe, from 59 to 69° N. However, this overall absence of latitudinal changes resulted from superposition of two opposing patterns: the percentage of leaves infested by eriophyoid mites decreased with latitude in *B*. *pubescens* and increased in *B*. *pendula*. These patterns were explained by mid-summer temperature in *B*. *pubescens* and by both mid-summer temperature and precipitation in *B*. *pendula*. Furthermore, these two patterns resulted from preference of these birch species by different species (or genotypes) of gall-making mites, which show different responses to climate. These findings in particular stress the need to properly identify host plants and herbivores in studies addressing their interactions. Assuming that the latitudinal patterns detected by our study are to a certain extent driven by climatic gradients, we suggest that climate warming may have opposite effects on gall mite infestation of the two co-occurring birch species, potentially affecting their competitive interactions in favour of *B*. *pendula* in subarctic forests and thus facilitating its range extension towards the North.

## Supporting Information

S1 Data Results of field censuses: numbers of leaves with different types of galls. (XLS)

S1 Fig Classification of leaf galls. (PDF)

S1 Table Coordinates of the study sites and a list of mite species collected at each site. (PDF)

S2 Table Latitudinal patterns (Spearman rank correlation coefficients with latitudes of study sites) in percentages of leaves with different types of galls by tree species. (PDF)

S3 Table Uncorrected pairwise genetic distances (%) with standard errors in parentheses between (regular font) and within (boldfaced) mite genotypes. (PDF)
