# Supplementary material for: Two Birch Species Demonstrate Opposite Latitudinal Patterns in Infestation by Gall-Making Mites in Northern Europe
Source: PLoS One. 2016 Nov 11;11(11):e0166641. doi: 10.1371/journal.pone.0166641 (PMC5105990; doi:10.1371/journal.pone.0166641)

Kozlov, M. V., Skoracka, A., Zverev, V., Lewandowski, M. and Zvereva, E. L. 2016. Two birch species demonstrate opposite latitudinal patterns in infestation by gall-making mites in Northern Europe.

S1 Figure. Classification of leaf galls (used in field counts).

Type 1. Red erineum (usually on the upper side of a leaf), which at low levels of infestation is not associated with deformation of leaf lamina.

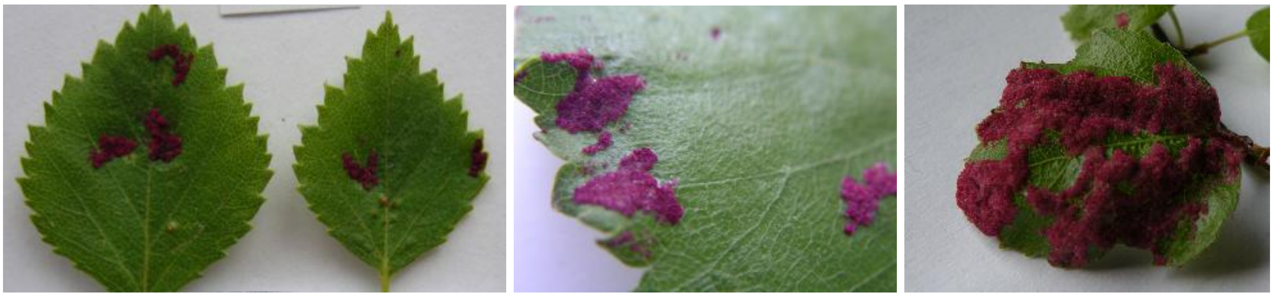

Type 2. Semitranslucent to white erineum (both on upper and lower sides of a leaf), which at low levels of infestation is not associated with deformation of leaf lamina.

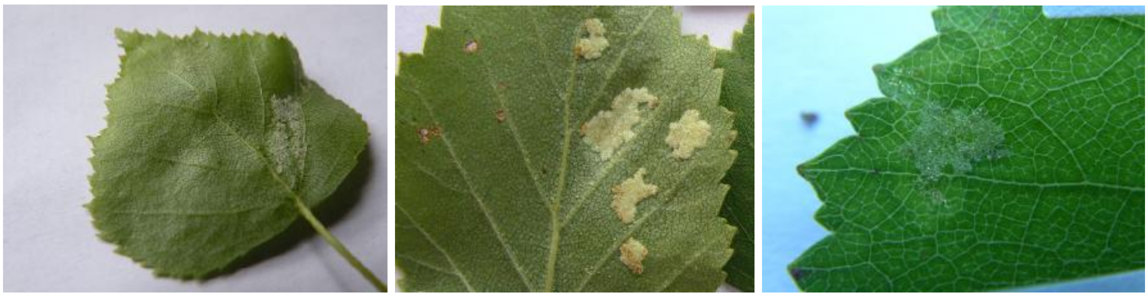

Type 3. Large tubercules with glossy to matt surface on the upper side, usually not associated with midvein, with white erineum on the lower side of a leaf.

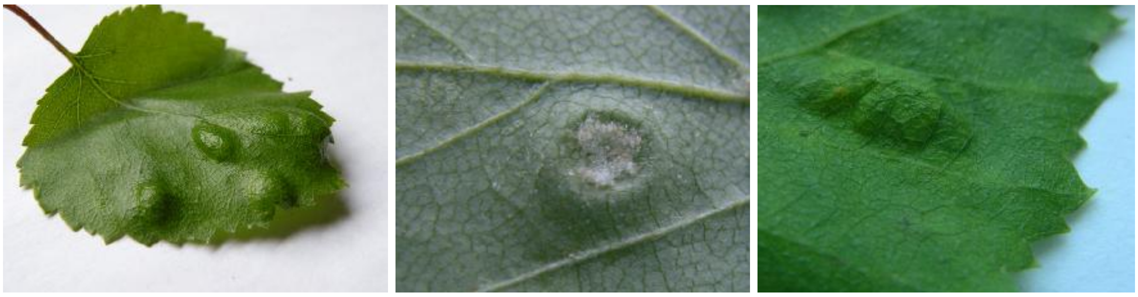

Type 4. Small tubercules with velvet surface on the upper side, usually not associated with midvein, with white erineum on the lower side of a leaf.

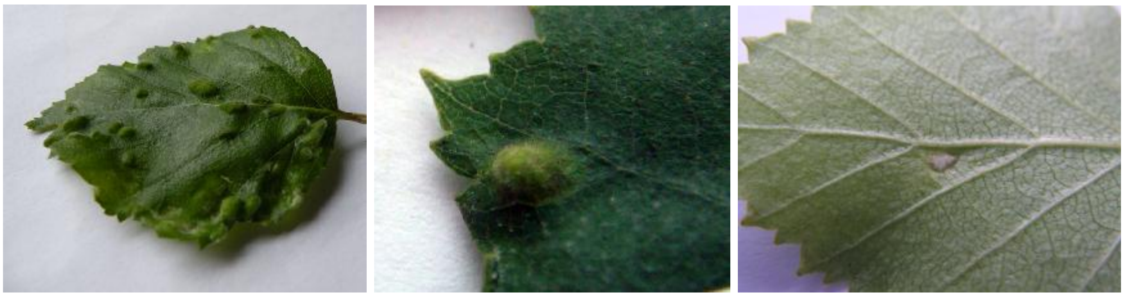

Type 5. Warty, pouch, or cephaloneon galls, green to red, on both sides of a leaf.

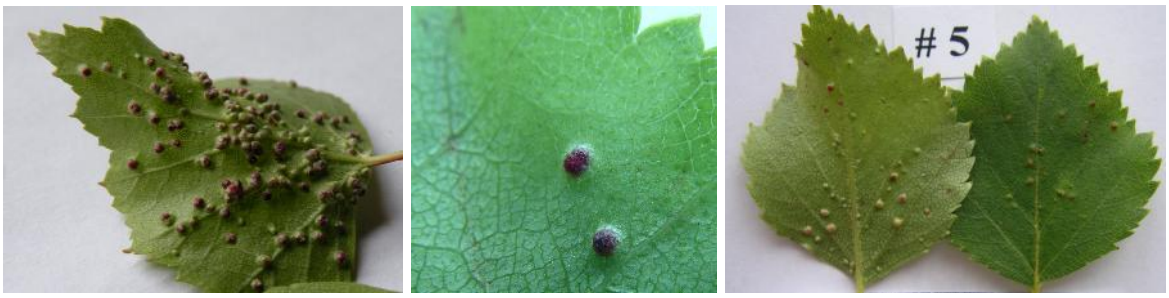

Type 6. Small tubercules with glossy surface on the upper side, located between midvein and the base of the second-order vein (vein angle galls), with white to ochreous brown erineum on the lower side of a leaf.

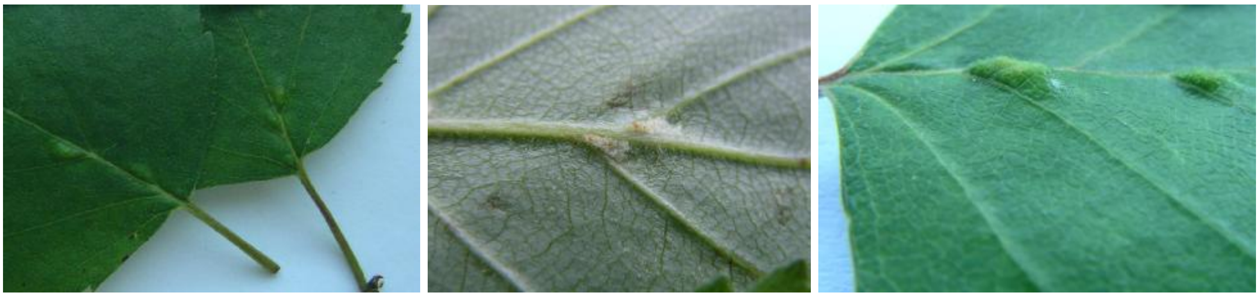

Supplement: S1 Fig — (PDF) [file pone.0166641.s002.pdf]
