# Supplementary material for: Two Birch Species Demonstrate Opposite Latitudinal Patterns in Infestation by Gall-Making Mites in Northern Europe
Source: PLoS One. 2016 Nov 11;11(11):e0166641. doi: 10.1371/journal.pone.0166641 (PMC5105990; doi:10.1371/journal.pone.0166641)
Supplement: S1 Table — (PDF) [file pone.0166641.s003.pdf]

Kozlov, M. V., Skoracka, A., Zverev, V., Lewandowski, M. and Zvereva, E. L. 2016. Two birch species demonstrate opposite latitudinal patterns in infestation by gall-making mites in Northern Europe.

S1 Table. Coordinates of the study sites and a list of mite species collected at each site.

Study site[a]	Country	Coordinates		Species of gall mites (identified from microscopic preparations)[b]					
		Latitude, N	Longitude, E	*A.lon.*	*A.rud.*	*A.fen.*	*A.lis.*	*A.vin.*	*E.lei.*
N60	Norway	59° 47' 34"	10° 45' 35"	+	+				+
N61	Norway	61° 04' 40"	11° 20' 46"	+					
N62*	Norway	62° 04' 19"	10° 42' 40"			+			+
N63	Norway	62° 59' 28"	10° 15' 50"						+
N64*	Norway	63° 54' 15"	11° 26' 25"	+	+	+			+
N65*	Norway	65° 04' 19"	13° 18' 15"	+					+
N66*	Norway	66° 02' 56"	13° 36' 32"	+					+
N67*	Norway	66° 59' 44"	15° 20' 22"						+
N68*	Norway	68° 02' 26"	15° 58' 32"		+				
N69*	Norway	68° 59' 30"	18° 30' 51"						+
F61	Finland	61° 00' 27"	23° 30' 42"						
F62	Finland	62° 00' 06"	25° 31' 30"						
F63	Finland	63° 03' 14"	25° 49' 33"						+
F64	Finland	63° 53' 48"	25° 47' 52"						+
F65*	Finland	64° 45' 47"	25° 34' 09"						
F66	Finland	65° 58' 32"	24° 39' 49"						
F67	Finland	66° 58' 49"	26° 19' 32"						
F68	Finland	67° 58' 35"	26° 50' 53"						
F69	Finland	69° 00' 41"	27° 00' 39"						
F70*	Norway	70° 04' 41"	27° 59' 22"						
R60	Russia	59° 58' 26"	32° 11' 46"						+
R61	Russia	61° 00' 03"	33° 03' 41"						+
R62	Russia	61° 58' 51"	34° 14' 27"		+				+
R63	Russia	63° 00' 07"	34° 22' 55"						+
R64	Russia	64° 01' 44"	34° 04' 11"						+

R65	Russia	65° 01' 25"	34° 00' 40"						+
R66	Russia	66° 01' 57"	32° 59' 13"		+				+
R67	Russia	66° 56' 04"	32° 12' 24"						+
R68	Russia	68° 01' 05"	32° 57' 07"						+
R69*	Russia	68° 52' 34"	33° 07' 42"						
A58.7	Russia	58° 40' 55"	40° 18' 35"		+				+
A59.5	Russia	59° 32' 17"	40° 25' 20"						+
A60.5	Russia	60° 29' 04"	41° 48' 50"				+	+	+
A61.1	Russia	61° 08' 22"	42° 11' 40"	+					
A61.9	Russia	61° 51' 49"	42° 38' 01"	+	+		+	+	+
A62.7	Russia	62° 41' 52"	42° 53' 35"	+					
A63.5	Russia	63° 30' 59"	41° 42' 55"	+	+				+
A64.2	Russia	64° 13' 25"	42° 37' 44"	+					+
A64.9	Russia	64° 54' 21"	43° 31' 03"						+
A65.6*	Russia	65° 34' 27"	44° 37' 35"	+	+				+
K59.1	Russia	59° 07' 31"	39° 11' 26"						
K60	Russia	59° 57' 11"	42° 37' 07"	+			+		+
K60.7	Russia	60° 43' 13"	46° 14' 51"						+
K61.3	Russia	61° 16' 35"	47° 04' 04"	+	+				+
K62	Russia	61° 55' 37"	50° 40' 47"						
K62.7	Russia	62° 38' 42"	51° 03' 52"		+				+
K63.2	Russia	63° 11' 32"	52° 38' 30"						
K63.7	Russia	63° 44' 14"	54° 12' 43"		+				+
K65.1*	Russia	65° 08' 33"	57° 16' 22"		+				+
K66*	Russia	66° 00' 16"	60° 20' 14"		+				

[a]*Betula pendula* absent in sites marked with an asterisk.

[b]Species of gall mites: A.lon. - *Acalitus longisetosus*; A.rud. - *Acalitus rudis*; A.fen. - *Aceria fennica*, A.lis.- *Aceria lissonota*, A.vin. - *Aceria vinosa*, E.lei. - *Eriophyes leionotus*; A. fen., A. lis, & A.vin were found on *Betula pubescens* only.
