# Supplementary material for: Two Birch Species Demonstrate Opposite Latitudinal Patterns in Infestation by Gall-Making Mites in Northern Europe
Source: PLoS One. 2016 Nov 11;11(11):e0166641. doi: 10.1371/journal.pone.0166641 (PMC5105990; doi:10.1371/journal.pone.0166641)
Supplement: S2 Table — (PDF) [file pone.0166641.s004.pdf]

Kozlov, M. V., Skoracka, A., Zverev, V., Lewandowski, M. and Zvereva, E. L. 2016. Two birch species demonstrate opposite latitudinal patterns in infestation by gall-making mites in Northern Europe.

S2 Table. Latitudinal patterns (Spearman rank correlation coefficients with latitudes of study sites) in percentages of leaves with different types of galls by tree species.

Gall type	Sampling period	N, 2011		F, 2008		R, 2008		R, 2009		R, 2010		R, 2011		A, 2010		K, 2009	
		pen	*pub*	*pen*	*pub*	*pen*	*pub*	*pen*	*pub*	*pen*	*pub*	*pen*	*pub*	*pen*	*pub*	*pen*	*pub*
		N=3	*N* =10	*N* =8	*N* =9	*N* =9	*N* =10	*N* =9	*N* =10	*N* =9	*N* =10	*N* =9	*N* =10	*N* =9	*N* =10	*N* =8	*N* =10
1	Early summer	-	-0.51	n.e.	n.e.	n.e.	n.e.	-	0.13	-	0.21	-	0.02	-	0.50	-	0.01
2	Early summer	-0.50	**-0.68**	n.e.	n.e.	n.e.	n.e.	-0.11	0.30	0.04	0.04	0.31	0.28	0.07	0.16	0.02	0.16
3	Early summer	-	0.38	n.e.	n.e.	n.e.	n.e.	0.32	-0.35	-	-	-	-	-	-	0.25	0.30
4	Early summer	-	-	n.e.	n.e.	n.e.	n.e.	**0.68**	0.10	-0.55	-0.53	-0.55	-0.52	-	-0.06	-0.43	-
5	Early summer	**-1.00**	-0.01	**0.79**	0.53	**0.68**	-0.01	-	0.41	0.28	-0.16	0.18	0.20	0.33	-	0.26	**0.64**
6	Early summer	-	0.52	n.e.	n.e.	n.e.	n.e.	-	-0.62	-0.28	**-0.83**	0.09	-0.51	-	0.02	-	-0.58
1	Late summer	-0.87	-0.50	n.e.	n.e.	n.e.	n.e.	-	-	-	0.17	-	0.32	-	0.18	-0.58	0.21
2	Late summer	**-1.00**	-0.08	n.e.	n.e.	n.e.	n.e.	0.38	0.12	0.13	0.20	**0.72**	0.38	0.48	0.45	0.31	0.61
3	Late summer	-	0.52	n.e.	n.e.	n.e.	n.e.	-	-	-	-0.06	-	-	-	-	-	-
4	Late summer	-	-0.18	n.e.	n.e.	n.e.	n.e.	-0.55	-	-0.18	-0.32	-	0.07	-	-	-	-
5	Late summer	**-1.00**	0.09	**0.71**	-0.14	0.45	0.34	0.33	0.06	0.31	0.07	-0.18	0.55	0.03	0.11	-0.33	-0.52
6	Late summer	-	-0.07	n.e.	n.e.	n.e.	n.e.	-	-0.28	-	**-0.72**	-	-0.62	-	-0.10	0.52	-0.60

Missing values indicate absence of a gall type in the particular data set (gradient × study year × sampling date × birch species); n.e. – not evaluated; coefficients significant at P = 0.05 are shown in bold; *pen*, *Betula pendula*; *pub*, *B. pubescens*; *N* = number of study sites within the gradient. For the description of gall types, consult Appendix 2.
