# Supplementary material for: Two Birch Species Demonstrate Opposite Latitudinal Patterns in Infestation by Gall-Making Mites in Northern Europe
Source: PLoS One. 2016 Nov 11;11(11):e0166641. doi: 10.1371/journal.pone.0166641 (PMC5105990; doi:10.1371/journal.pone.0166641)
Supplement: S3 Table — (PDF) [file pone.0166641.s005.pdf]

Kozlov, M. V., Skoracka, A., Zverev, V., Lewandowski, M. and Zvereva, E. L. 2016. Two birch species demonstrate opposite latitudinal patterns in infestation by gall-making mites in Northern Europe.

S3 Table. Uncorrected pairwise genetic distances (%) with standard errors in parentheses between (regular font) and within (boldfaced) mite genotypes.

Species	Genotype	Gall type	A	B	C	D	E	F	G	H
Acalitus longisetosus	A	1	**0.0 (0.0)**							
	B	2	21.4 (1.6)	**0.1 (0.1)**						
Acalitus rudis	C	1-3	36.4 (1.9)	32.1 (1.8)	**0.0 (0.0)**					
Aceria fennica	D	2-3	37.3 (2.0)	33.6 (1.9)	10.8 (1.2)	**0.3 (0.2)**				
Aceria lissonota	E	6	34.8 (1.9)	32.8 (1.9)	16.2 (1.4)	15.5 (1.4)	**0.1 (0.1)**			
Eriophyes leionotus	F	3-4	22.5 (1.5)	15.2 (1.4)	30.1 (1.8)	32.3 (1.8)	30.6 (1.7)	**0.0 (0.0)**		
	G	5	32.5 (1.8)	29.6 (1.9)	29.3 (1.8)	30.5 (1.9)	25.8 (1.8)	23.0 (1.7)	**0.0 (0.0)**	
	H	6	34.3 (1.8)	30.8 (1.8)	30.6 (1.9)	31.6 (1.9)	28.3 (1.8)	24.2 (1.7)	16.7 (1.5)	**0.1 (0.0)**
